# Effect of Reducing the Size and Number of Faces of Polyhedral Specimen on Wood Characterization by Ultrasound

**DOI:** 10.3390/ma16134870

**Published:** 2023-07-07

**Authors:** Cinthya Bertoldo, Geise Aparecida Pereira, Raquel Gonçalves

**Affiliations:** Laboratory of Nondestructive Testing—LabEND, School of Agricultural Engineering—FEAGRI, University of Campinas—UNICAMP, Av. Cândido Rondon, 501-Barão Geraldo, Campinas 13083-875, Brazil; eng.geisepereira@gmail.com (G.A.P.); raquelg@unicamp.br (R.G.)

**Keywords:** longitudinal modulus of elasticity, shear modulus, Poisson’s ratio, growth rings’ inclination

## Abstract

The complete characterization of wood, with the determination of the 12 elastic constants that represent its orthotropy, is greatly relevant in applications employing structural calculation software programs. Ultrasound allows for such characterization with relative simplicity when compared to other methods. The polyhedron is considered the most appropriate specimen format for allowing the 12 constants to be calculated with a single specimen, and the traditionally used one is the 26-sided polyhedron, which, to be produced manually with more precision in directing the main directions of the wood, needs larger faces. The accuracy of this technique tends to be reduced when increasing the growth rings’ inclination since the waves deviate from the main directions of orthotropy. This research aimed to verify whether it is possible to reduce the polyhedra dimension without affecting the results of the elastic parameters obtained in wood characterization by ultrasound. The results indicate that the dimension of the polyhedron can be reduced without prejudice to the results of the elastic parameters obtained by the ultrasound test and that, in the manual production process of the specimen, the best way to make this reduction is to eliminate the faces unused in the test, changing the polyhedron to 18 faces instead of 26. Reducing the number of faces simplifies the manufacturing process and thus increases the possibility of producing specimens with straighter growth rings and better-directed symmetry axes.

## 1. Introduction

Wood is a viable material that is suitable for use in different applications. In civil construction, it has been highly recommended worldwide because of its sustainability since it is a renewable natural resource with low energy consumption and a great contribution to reducing greenhouse gases [1,2].

Currently, there are structural calculation programs that allow for precise dimensioning of structures, even the most complex ones. However, the accuracy of these tools depends on the insertion of data in a complete and coherent way with the type of material (isotropic, orthotropic, and transversely isotropic), making the acquisition of these data essential. In addition to civil construction, wood has great importance in the manufacture of musical instruments, which is another example of an area for which knowledge of elastic properties is essential [3,4,5,6,7,8,9]. Nevertheless, as wood is a complex material due to its anisotropic and heterogeneous characteristics, obtaining knowledge of its mechanical properties is still a challenge [10,11].

By reducing the dimensions of specimens, it is possible to obtain more rectilinear growth rings and, thus, get closer to the condition of orthotropy with three main axes of symmetry (longitudinal—L, radial—R, and tangential—T) that respond differently to the action of loads. The L axis is parallel to the direction of the grain (fiber or tracheid); the R axis is perpendicular to the grain in the direction of the radius; and the T axis is perpendicular to the grain but tangents the growth rings. Thus, for the complete characterization of this material, one must determine twelve elastic constants (nine of them independent): three longitudinal moduli of elasticity (E_L_, E_R_, E_T_), three shear moduli of elasticity (G_LR_, G_LT_, G_RT_), and six Poisson ratios (μ_LR_, μ_RL_, μ_RT_, μ_TR_, μ_LT_, μ_TL_).

Ultrasound technology allows one to determine, in a non-destructive way, all the elastic constants of orthotropic materials, such as wood, and its theoretical framework is discussed by Bucur [12], Musgrave [13], and Hearmon [14]. Over the years, with the development of equipment, several studies have been conducted corroborating the effectiveness and accuracy of ultrasound technology in the characterization and inference of wood properties [15,16,17,18,19,20,21,22,23]. For the complete characterization of wood with ultrasound, it is possible to use several specimen formats, such as spherical [10,24], prismatic [3,20,25,26,27], polyhedral [12,20,22], and multifaceted discs [12,20]. Polyhedral specimens with 26 faces were initially proposed by Francóis [28] and polyhedral specimens with 18 faces were proposed by Arts [28,29].

In the process of the complete characterization of wood by ultrasound, some authors report difficulties in determining the terms outside the main diagonal of the stiffness matrix [12,20] since a distortion of the wavefront, caused by the inclination of the wood growth rings, can be observed. According to Bucur and Archer [27], reducing the size of the specimen favors the approximation of orthotropy since the curvature of the growth rings can be neglected, while in larger sections, the curvatures of the growth rings are significant [30]. On the other hand, the reduction in the specimen must be compatible with the maintenance of plane wave propagation in infinite media, and for this, one must make the reduction in dimensions compatible with the frequency of the transducer used [12]. Considering the identification of interferences in the inclination of the growth rings in the results of ultrasound characterization and the greater feasibility of obtaining specimens with more rectilinear growth rings, reducing the size of the specimen, this research aimed to verify whether the reduction in the polyhedral specimen compared to the traditionally used one (26 faces starting from a 70 mm edge cube) changes the results of the elastic parameters obtained in the characterization of wood by ultrasound. To achieve the objectives, the experimental design involved the determination of the elastic parameters of three wood species using polyhedral specimens of 26 faces and dimensions traditionally used in the literature (starting from 70 mm edge cubes), which were considered as references, as well as polyhedra of 26 faces of reduced dimension and polyhedra of 18 faces, both starting from edge cubes with approximately half the traditional polyhedron dimension (≅35 mm). To minimize interference caused by other parameters, dimension reductions were made to continue guaranteeing the theoretical framework of wave propagation in infinite media.

Thus, the main novelty of this study is the analysis of the behavior of the results of the elastic characterization of wood by ultrasound using specimens in a polyhedral format with reduced dimensions and fewer faces than those traditionally used. This analysis offers a safer basis for the modification of a traditionally used and known specimen and, as a consequence, reduces the amount of material used in the manufacture of specimens and simplifies the process of producing polyhedra. The simplification of the process, in turn, allows for an increase in the accuracy of the specimen’s production with better-directed growth rings.

## 2. Materials and Methods

**Figure 1:**
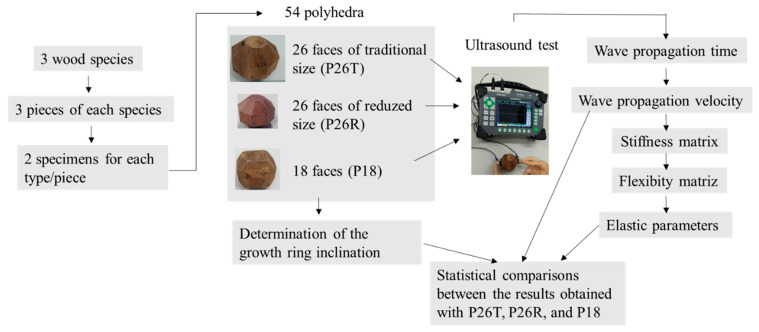
Experimental Design Scheme.

The experimental scheme of the research, presented in detail in this topic, is shown in Figure 1.

### 2.1. Material

Three wood species were selected for the tests: *Apuleia leiocarpa*, *Dinizia excelsa*, and *Cedrelinga catenaeformis*. The choice of species was made to cover a range of densities that would allow for greater generalization of the results.

For each species, three pieces of 0.10 × 0.20 × 1.0 m³ were obtained, which were purchased with moisture already around 12% (Table 1). The pieces had their weight and volume measured for the calculation of densities (Table 1) and monitoring of moisture by contact measurement equipment (Merlin, PM1-E, Tumeltsham, Austria).

For wood, a material with great variability within the same species and even within the same tree, comparisons with samples from other authors that aim at the validation of tests are made only based on the order of magnitude of the results. According to the Institute of Technological Research [31], *Apuleia leiocarpa* and *Dinizia excelsa* have average apparent densities (with 12% moisture) of 830 kg·m^−3^ and 1090 kg·m^−3^, respectively, which are values with mean differences of around 2.5% from those obtained in this study. In the case of *Cedrelinga catenaeformis*, the difference was 22% compared to that obtained by Dias and Lahr [32], of 566 kg·m^−3^. This species was also the one with the highest density variability between the pieces (Table 1). Despite the greater difference obtained for *Cedrelinga catenaeformis*, the order of magnitude of the densities obtained in this research is compatible with the one obtained by other authors.

### 2.2. Acquisition of Polyhedral Specimens

**Figure 2:**
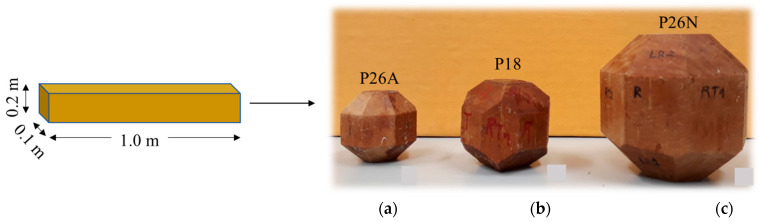
Schematic representation of the specimens made from the pieces. Two polyhedra of each type were made from each piece. Polyhedron with 26 faces of reduced dimension (P26A—(**a**)); polyhedron with 18 faces (P18—(**b**)), polyhedron with 26 faces of traditional dimension (P26N—(**c**)).

The polyhedra that composed the research sample and that were used in the ultrasound tests were obtained from the 0.10 × 0.20 × 1.0 m^3^ pieces (Table 1). For each of the species, 6 polyhedra of each shape and dimension adopted in the research were made (Figure 2), totaling 54 polyhedra, including 18 polyhedra with 26 faces (P26N) with dimensions traditionally used in the literature, 18 polyhedra with 26 faces of reduced dimension (P26A), and 18 polyhedra with 18 faces (P18).

**Figure 3:**
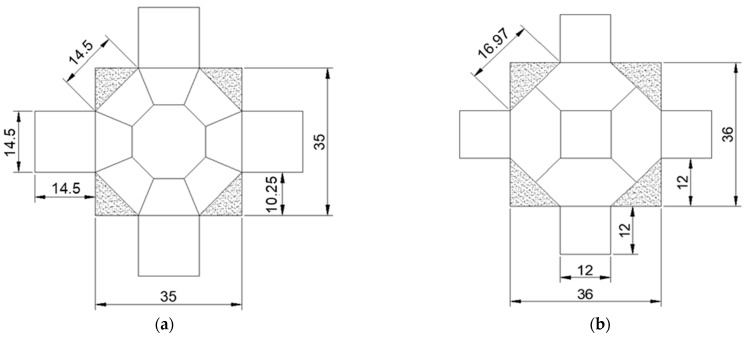

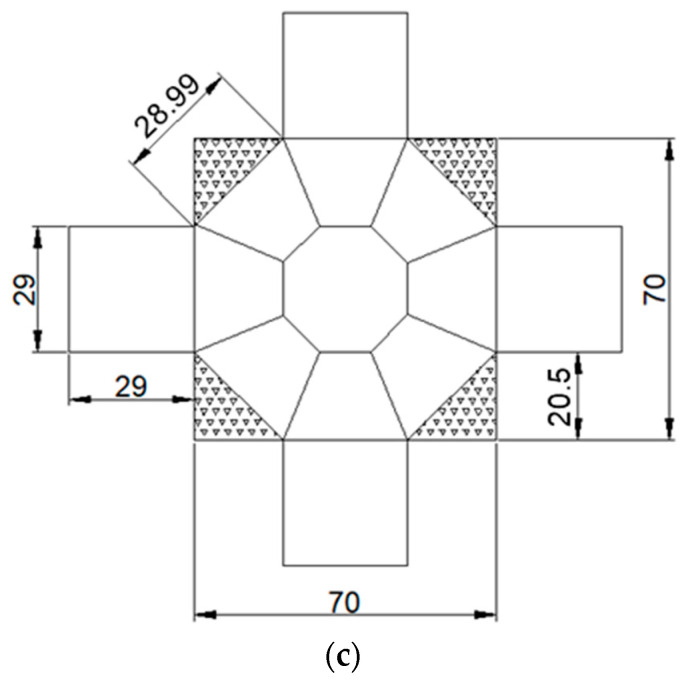
Top view of polyhedra with 26 faces of reduced dimensions (**a**), polyhedra with 18 faces (**b**), and polyhedra with 26 faces of traditionally used dimensions ((**c**) adapted from Vasques et al. [22]). Dimensions in mm.

The polyhedra were taken from cubes oriented in the three main directions of the wood (L or 1, R or 2, and T or 3), with the growth rings as rectilinear and parallel as possible in relation to the edge of the cube. The polyhedron with 26 faces of traditionally used dimensions [17,18,19,20,22] was obtained from a 70 mm edge cube (Figure 3c). The polyhedron with 26 faces and reduced dimensions was made from a 35 mm edge cube (Figure 3a), half the size of the cube edge (70 mm) that originated the traditionally used polyhedron (Figure 3c). The dimension of the cube edge that originated the polyhedron with 18 faces (Figure 3b) was adopted to maintain, as the minimum dimension of the faces, the diameter of the 1.0 MHz frequency transducer (12 mm), ensuring that the transducer could be circumscribed to the face of the polyhedron, avoiding the wall effect during wave propagation [12]. The 1.0 MHz frequency transducer was adopted as a reference for the proposal of the dimensions of the specimens because this frequency level is considered the maximum that allows for the maintenance of a wavelength compatible with the dimensions of the anatomical elements of the wood, minimizing signal attenuation problems [12].

### 2.3. Measurement of the Growth Ring Inclination on the Face of the Polyhedron

**Figure 4:**
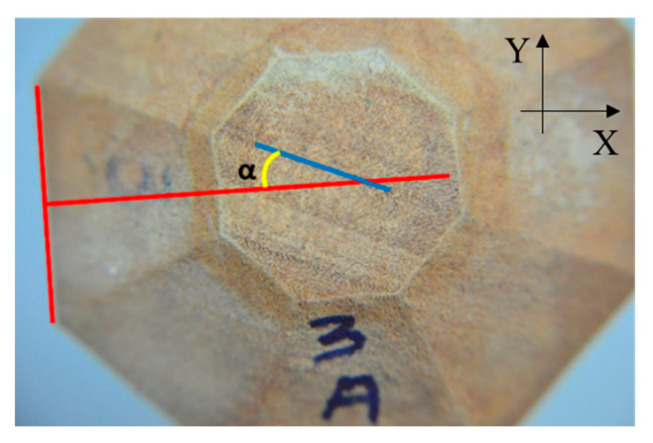
Direction of the growth ring (blue line) and radial (R) and tangential (T) directions parallel to the Y and X axes (red lines), with the red line crossing the cross section indicative of the direction of propagation of the ultrasound wave.

Considering that the reduction in the dimension of the polyhedra is proposed to test the hypothesis that it allows for a reduction in the inclination of the growth rings compared to the radial or tangential face and that this reduction minimizes wave dispersion effects, improving the purest propagation in the axes or planes, the inclination of the rings was measured. To measure the inclination of the growth rings, it was first necessary to sand the cross section (RT) of each polyhedron until it was possible to clearly identify the rings, using sandpaper weights of 120 and 400. Initially, the samples were sanded in an orbital sander (DeWalt, DWE6411, Uberaba-MG, Brazil), and then, to allow a finer finish, a polisher (Panambra, DP-10, São Paulo-SP, Brazil) was used. The cross section (RT) of the polyhedra was then photographed using a camera (NIKON D7000 with AF-S DX VR Zoom-Nikkor 18–105 mm ƒ 3.5–5.6 lens) fixed on a tripod. The samples were positioned so that the images had the radial and tangential directions parallel to the Y and X axes (Figure 4). The images were then opened in an app (Paint) where the direction of the growth ring (blue line in Figure 4) and the radial (R) and tangential (T) directions (red lines in Figure 4) were demarcated, the red line crossing the cross section being indicative of the direction of propagation of the ultrasound wave (Figure 4). The lines (blue and red) were used to determine the angle α (Figure 4) between the direction of the growth rings and the direction of propagation of the ultrasound wave using the ImajeJ software (1.53t).

### 2.4. Ultrasound Tests on Polyhedra

**Figure 5:**
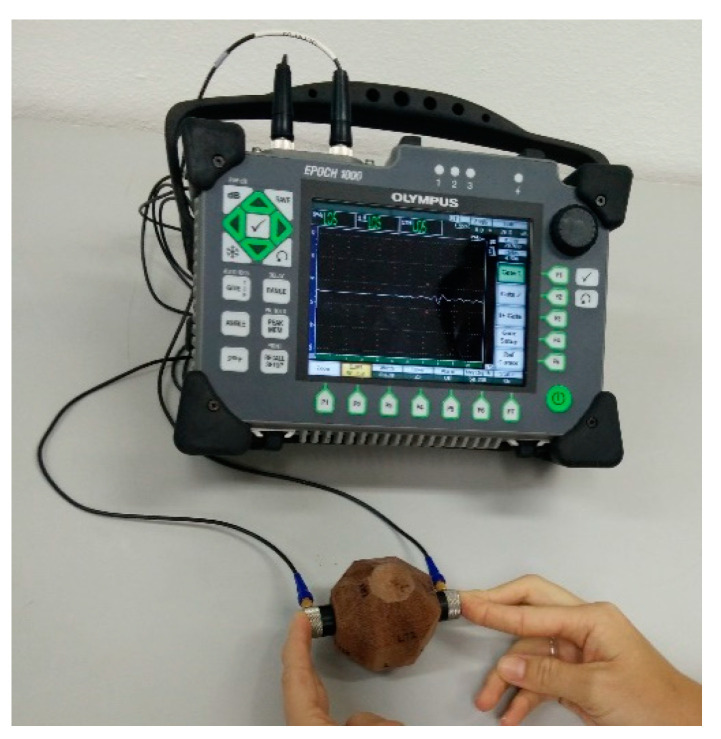
Ultrasound test on polyhedra for wood characterization.

Tests on polyhedral specimens were performed with ultrasound equipment (EP1000, Olympus, Waltham, MA, USA) with 1 MHz frequency compression and shear wave transducers (Figure 5). The coupling between the transducer and the material to be tested is a factor of great importance for the accuracy of the results, and the use of viscous material is indicated to eliminate the air between the transducers and the surface of the evaluated specimen. The coupler also has the function of reducing the impedance difference in the wave passage to the material under inspection, minimizing the attenuation of the signal as a function of wave deviation (Snell’s law); this research adopted starch glucose, which, according to Gonçalves et al. [17], presents good results for the shear wave in wood.

### 2.5. Calculation of Wave Propagation Velocities

For the calculation of longitudinal velocities (V_11_, V_22_, and V_33_), Equation (1) was used, in which the path length corresponds to the distance between the faces of the transducers and the wave propagation times in directions 1 (longitudinal), 2 (radial), and 3 (tangential), respectively, are obtained using the longitudinal transducer.

(1) $$V=\frac{L}{t}*10^{6}$$

where V = velocity of wave propagation in a given direction (m·s^−1^); L = wave path length (m); t = wave propagation time in a given direction (µs).

Considering the same directions but with the use of the shear transducer, the transversal velocities (V_12_, V_13_, V_21_, V_31_, V_32_, and V_23_) were calculated by obtaining the wave path time (t_12_, t_13_, t_21_, t_31_, t_32_, and t_23_) with propagation in a certain direction (first numerical index) and its polarization in the perpendicular direction (second numerical index).

For the determination of the velocities corresponding to the wave propagation outside the axes of symmetry, the wave propagation time was obtained using shear transducers, but on the faces representing axes inclined 45° with respect to each plane.

### 2.6. Calculation of Stiffness [C] and Flexibility [S] Matrices

**Figure 6:**
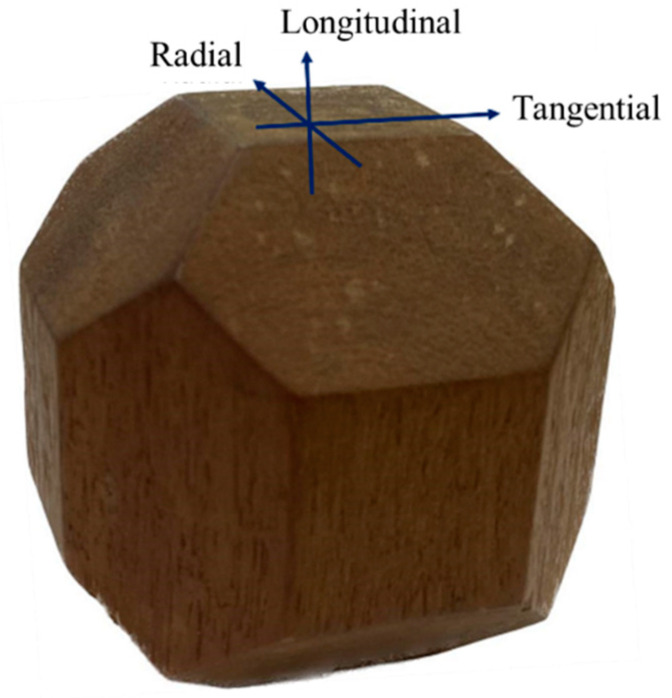
Representation in the polyhedron of the three main axes of symmetry of wood.

With the velocity data, the stiffness matrix [C] (Equation (2)) was determined using Christoffel’s equations (Equations (3)–(6)). The density values in the equations were those obtained for the pieces used to make the polyhedra (Table 1). The stiffness coefficients of the main diagonal (C_11_, C_22_, C_33_, C_44_, C_55_, and C_66_) were obtained by the Christoffel equation (Equation (3)) with the longitudinal velocities (V_11_, V_22_, and V_33_) propagating and polarizing in the direction of the main axes of symmetry (longitudinal, radial, and tangential: Figure 6). To calculate coefficients C_44_, C_55_, and C_66_, the velocities whose wave propagation times on the axes were obtained with the shear transducer were used, with wave propagation on one axis and its polarization on the perpendicular axis, with the numbering related to the axes (propagation/polarization) having the following nomenclature: 44 = propagation on axis 2 and polarization on axis 3 or propagation on axis 3 and polarization on axis 2 (plane 2–3); 55 = propagation on axis 1 and polarization on axis 3 or propagation on axis 3 and polarization on axis 1 (plane 1–3); and 66 = propagation on axis 1 and polarization on axis 2 or propagation on axis 2 and polarization on axis 1 (plane 1–2). In the same way (by solving the Christoffel equation), the 3 terms outside the diagonal (C_12_, C_13_, and C_23_) can be obtained. For this, the propagation must occur inclined to the axes of symmetry (45° in this research). The general formulas, deduced from the Christoffel tensor for determining the constants in planes 1–2 (LR), 2–3 (RT), and 1–3 (LT), are given by Equations (4)–(6).

(2) $$\left[C\right]=\begin{array}{cccccc} C_{11} & C_{12} & C_{13} & 0 & 0 & 0\\ C_{12} & C_{22} & C_{23} & 0 & 0 & 0\\ C_{13} & C_{23} & C_{33} & 0 & 0 & 0\\ 0 & 0 & 0 & C_{44} & 0 & 0\\ 0 & 0 & 0 & 0 & C_{55} & 0\\ 0 & 0 & 0 & 0 & 0 & C_{66} \end{array}$$

(3) $$C_{\mathrm{ii}}=\rho \cdot V_{\mathrm{ii}}{}^{2}$$

where i = 1, 2, 3, 4, 5, and 6; ρ = density of the material (kg·m^−3^); V = velocity of wave propagation in the considered direction (m·s^−1^).

(C_12_ + C_66_) n_1_n_2_ = [(C_11_ n_1_^2^ + C_66_ n_2_^2^ − ρ V_α_^2^) (C_66_ n_1_^2^+ C_22_ n_2_^2^ − V_α_^2^)]^½^ (4)

(C_23_ + C_44_) n_2_n_3_ = [(C_22_ n_2_^2^ + C_44_ n_3_^2^ − ρ V_α_^2^) (C_44_ n_2_^2^ + C_33_ n_3_^2^ − ρ V_α_^2^)]^½^ (5)

(C_13_ + C_55_) n_1_n_3_ = [(C_11_ n_1_^2^ + C_55_ n_3_^2^ − ρ V_α_^2^) (C_55_ n_1_^2^ + C_33_ n_3_^2^ − ρ V_α_^2^)]^½^ (6)

where α = angle (45°); n_1_ = cos α, n_2_ = sin α, and n_3_ = 0 (α is taken with respect to axis 1) (plane 1–2) for Equation (4); n_1_ = cos α, n_3_ = sin α, and n_2_ = 0 (α is taken with respect to axis 1) (plane 1–3) for Equation (5); n_2_ = cos α, n_3_ = sin α, and n_1_ = 0 (α is taken with respect to axis 2) (plane 2–3) for Equation (6).

By inverting the matrix [C]^−1^, it was possible to obtain the flexibility matrix [S] (Equation (7)), which is associated with the elastic parameters of the material (longitudinal moduli of elasticity—E, shear moduli of elasticity—G, and Poisson’s ratio—υ).

(7) $$[S]=\left[\begin{array}{cccccc} \frac{1}{E_{1}} & -\frac{\upsilon_{21}}{E_{2}} & -\frac{\upsilon_{31}}{E_{3}} & 0 & 0 & 0\\ -\frac{\upsilon_{12}}{E_{1}} & \frac{1}{E_{2}} & -\frac{\upsilon_{32}}{E_{3}} & 0 & 0 & 0\\ -\frac{\upsilon_{13}}{E_{1}} & -\frac{\upsilon_{23}}{E_{2}} & \frac{1}{E_{3}} & 0 & 0 & 0\\ 0 & 0 & 0 & \frac{1}{G_{23}} & 0 & 0\\ 0 & 0 & 0 & 0 & \frac{1}{G_{13}} & 0\\ 0 & 0 & 0 & 0 & 0 & \frac{1}{G_{12}} \end{array}\right]$$

### 2.7. Verification of Basic Aspects of Wave Propagation in Infinite Media

As we used polyhedral specimens of reduced dimensions (18 faces and 26 faces reduced), it is important to analyze whether the theoretical aspects regarding wave propagation in infinite media are being met. For this propagation to occur in infinite media, the distance between transducers (dimension of the piece in the direction of wave propagation) must be a few times greater than the wavelength (λ). For wood, some researchers [12,33,34] indicated values from 2 λ to 5 λ for this condition to be met. The minimum dimension of the piece is linked to the frequency of the transducer (f) and to the propagation velocity of the wave in the material (V) since λ is given by the ratio between these two parameters (λ = V/f). When this theoretical condition is violated, the results of the elastic parameters obtained by ultrasound can be affected [12,26,34].

**Figure 7:**
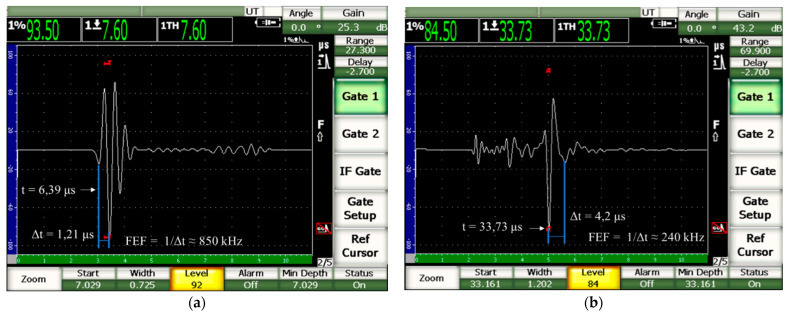
Example of a screen for capturing the compression wave in the longitudinal direction (**a**) and the shear wave in the radial–tangential plane (**b**) with calculations of the effective frequencies (FEF).

Because of the attenuation of the wave in the wood, the effective frequency (FEF) is different from the nominal frequency of the transducer (f), which, in the case of this research, is 1 MHz. To obtain the effective frequency (FEF), one must obtain the time difference (Δt) between two successive peaks of the wave (FEF = 1/Δt) and therefore the signal must be evaluated (example in Figure 7). Considering the frequency of the transducer and the dimensions of the specimens adopted in this research, it was found that, even in the most critical cases (shorter path length and higher velocity), at least two complete waves passed through the material (Table 2), thus meeting the theoretical conditions of infinite media.

### 2.8. Analysis of Results

The results of the inclination of the growth rings, wave propagation velocities (longitudinal and shear) in different directions and planes, and elastic parameters were initially evaluated for normality to ensure the feasibility of using parametric statistics. The multiple range test was used for the comparisons of the results for the traditional polyhedra with 26 faces, the reduced polyhedra with 26 faces, and the polyhedra with 18 faces. 

## 3. Results and Discussions

### 3.1. Inclination of Growth Rings on RT Faces of Polyhedra

For all species studied, the average inclinations of the growth rings were lower for P18, differing more from the P26A samples, which in general presented higher values (Table 3). The manufacturing process of the polyhedrons can explain this result, since the process is much simpler for the execution of the 18 faces than the 26 faces, improving the conditions for the cut to contemplate greater accuracy for the main directions. In the case of P26N, the fact that the piece is larger also facilitates the manufacturing process, but it is more difficult to obtain straighter growth rings. Finally, the execution of the reduced P26A is the most complex because it associates the difficulty of working on the machine with a very small piece with the difficulty of producing the 26 faces. These conditions explain why reducing the number of faces was more effective in reducing the inclination of the growth rings than dimension reduction.

### 3.2. Propagation Velocities of Ultrasound Waves

Considering theoretical aspects of wave propagation in wood [13,16,20], it is expected that V_11_ > V_22_ > V_33_, V_66_ > V_55_ > V_44_, and V_12 45°_ > V_13 45°_ > V_23 45°_. In the longitudinal direction of the wood, parallel to the fiber or tracheid (direction 1 or L), the continuity of the anatomical element allows the propagation of waves with higher velocity [12]. In the radial direction, the radiuses, because they are less continuous, induce velocities much lower than those of the longitudinal direction but still slightly higher than the propagation in the tangential direction, in which there is no continuously organized anatomical structure for its path [12]. According to Gonçalves et al. [20], the observed consistency of this relationship for all species analyzed and for the different formats of specimens evaluated corroborates the effectiveness and coherence of the method applied in the polyhedra. This behavior of the velocities was verified for all the specimens tested in this research, except for one of the specimens of the species *Dinizia excelsa*, which, with P26N, presented V_44_ with a value greater than V_55_, and thus it was the only value eliminated from the statistical analysis. All other values, besides behaving coherently in terms of the theoretical framework, had normal distributions, validating the application of parametric statistical methods. The mean values (Table 4, Table 5 and Table 6) also showed behavior of magnitudes of velocities consistent with the theoretical framework of wave propagation in wood for all types of specimens.

For all species, in 96% of cases, the velocity values obtained for P26A were statistically equivalent to P26N (Table 4, Table 5 and Table 6), while in 85% of cases, the equivalence was obtained for P18, indicating, once again, the greater influence of the change in the number of faces in the polyhedral specimen on the propagation velocities of the waves than their size. The only case of differences between longitudinal velocities occurred in the longitudinal direction in the species *Cedrelinga catenaeformis*, for both P26A and P18 (Table 6). In the rest of the cases, the differences were all for P18 and in the transversal velocities in the axes (V_44_) of species *Dinizia excelsa* (Table 5) or off-axis (V_13 45°_) of species *Apuleia leiocarpa* (Table 5) and V_23 45°_ of species *Dinizia excelsa* (Table 5).

It is important to highlight that, according to the methodology adopted for the removal of the specimens, for each species, three pieces were used, from which two specimens of each type were obtained, thus totaling the six specimens of each type that constituted the sample. The base material from which the specimens were taken presented natural variability, verified by the differences between densities observed between the pieces of the same species (Table 1). Thus, in addition to the expected influence of the variation in the inclination of the growth rings, the natural variability of the wood cannot be ruled out as one of the causes of the differentiation of velocities. Therefore, it is important to verify whether the differences in velocities obtained in the different types of polyhedra were sufficient to cause differences in elastic parameters.

### 3.3. Elastic Parameters Obtained with the Different Polyhedra

The mean values of the elastic parameters obtained from the ultrasound test in this research (Table 7, Table 8 and Table 9) are within the expected range for wood. *Apuleia leiocarpa* was characterized using ultrasound tests in prisms [17] and in polyhedra with 26 faces of traditional dimensions [20], with results of the same order of magnitude as those obtained for the elastic parameters in this research. As in the results of the literature [10,17,20,22], the greatest variabilities were verified by Poisson’s ratio, which is obtained from shear waves with propagation outside the symmetry axes, a condition in which the greatest inaccuracies of the method are verified. However, the values of Poisson’s ratio obtained in this research did not have values equal to or greater than 1.0, as in those obtained by Bucur [12] and Ozyhar et al. [35]. Bucur [12] shows that, although not usual, considering that the orthotropy of wood is a simplification, values greater than 1.0 could occur, especially for the coefficients ν_TR_ and ν_LT_. Thus, the elastic parameters obtained using polyhedral specimens of altered dimensions or shape indicated values compatible with those obtained in the literature and also with those expected considering the acoustic and mechanical properties of the wood.

In about 93% of the cases, no statistical difference was found between the elastic parameters obtained and the reduction in the dimensions of the polyhedra, either with 26 or 18 faces (Table 7, Table 8 and Table 9). The longitudinal velocity had been affected in the species *Cedrelinga catenaeformis* with the reduction in the specimen (Table 6), both for 26 and 18 faces, but this interference was not reflected in the longitudinal modulus of elasticity for P18 (Table 9). The shear modules in the RT and RL planes were affected by the reduction in the sided specimens (26 to 18) for the species *Dinizia excelsa* (Table 8), and the Poisson ratio was affected in the TR plane only for the 18-sided specimen in the species *Apuleia leiocarpa* (Table 7). This low incidence of differences in general terms (about 7%) indicates that the reduction did not significantly alter the results, compared to those obtained with the traditional specimen.

According to Kohlhauser and Hellmich [26], when the relationship between the sample length (wave propagation direction) and the radius of curvature of the growth rings approaches zero, the influence of growth ring curvature on ultrasound measurements can be disregarded. In general, for P18, the mean inclination of the growth rings was of the order of 3°, a value closer to the non-influence condition described by Kohlhauser and Hellmich [26] when compared to P26N (of the order of 7°) and P26A (of the order of 10°). Reducing the inclination of the growth rings tends to increase the accuracy of the elastic parameters since it enables purer cross-sectional directions (radial and tangential). Bucur and Archer (1984) used 16 mm edge cubes for ultrasound characterization of six wood species and argued that the smaller size of the specimen allows a better approximation of orthotropy since the curvature of the growth rings can be neglected.

However, in the case of this research, it is important to highlight that, in some cases (Table 7 and Table 9), the values of shear modules and, especially, of Poisson’s ratios presented high values of coefficient of variation for the specimens with reduced dimensions, which indicates that the reduction was not as effective as expected in reducing, by reducing the inclination of the growth rings, the variability of the results obtained by the method. In addition, high coefficient of variation values may have influenced, in some cases, the statistical equivalence of the results.

## 4. Conclusions

The results of this research allow us to conclude that it is possible to reduce the dimension or the number of faces of a polyhedric specimen without affecting the results of the elastic parameters of wood obtained by ultrasound testing. This result is important not only for the manual process used in this research for producing the specimens but also for more automated processes that could be adopted. In manual production processes of polyhedral specimens, reducing the number of faces of the polyhedron from 26 to 18 simplifies the manufacturing process, improving the conditions for the specimen to be produced with reduced dimensions and, even so, with better-directed growth rings, which, according to the bases of the ultrasound test, allow for an increase in the accuracy of the obtained elastic parameters since it enables purer wave propagation cross-sectional directions (radial and tangential).

## Figures and Tables

**Table 1:** Mean values and coefficients of variation (in parentheses) of the moisture content and apparent densities of each species.

Species	Moisture (%)	Density (kg·m^−3^)
*Apuleia leiocarpa*	10.6 (4.0%)	802 (10.6%)
*Dinizia excelsa*	11.6 (1.2%)	1083 (2.5%)
*Cedrelinga catenaeformis*	12.6 (0.6%)	501 (14.5%)

**Table 2:** Number of wavelengths propagating by the three types of specimens (P26N, P26A, and P18) considering the different directions/planes of wave propagation (example for species *Apuleia leiocarpa*).

Direction or Plane of Propagation	P26N	P26A	P18
11 (propagation and polarization of the compression wave in the longitudinal direction)	8.6	5.3	5.7
22 (propagation and polarization of the compression wave in the radial direction)	17.6	10.7	11.7
33 (propagation and polarization of the compression wave in the tangential direction)	18.1	11.1	11.4
66 (shear wave propagation in the longitudinal direction and polarization in the radial direction and vice versa)	24.1	4.9	2.9
55 (shear wave propagation in the longitudinal direction and polarization in the tangential direction and vice versa)	21.2	7.6	5.8
44 (shear wave propagation in the radial direction and polarization in the tangential direction and vice versa)	12.6	8.0	9.4
12_45° (in the inclined faces: shear wave propagation in the longitudinal direction and polarization in the radial direction and vice versa)	12.3	9.4	11.5
13_45° (in the inclined faces: shear wave propagation in the longitudinal direction and polarization in the tangential direction and vice versa)	10.2	4.8	7.9
23_45° (in the inclined faces: shear wave propagation in the radial direction and polarization in the tangential direction and vice versa)	15.8	7.8	12.1

**Table 3:** Mean values and coefficients of variation (in parentheses) of the angles of inclination of the growth rings on the RT faces of the traditional polyhedra (P26N), reduced polyhedra (P26A), and polyhedra with 18 faces (P18) in the different species and overall mean regardless of the species.

	P26N	P26A	P18
*Apuleia leiocarpa*	13.0° (81%) b	12.9° (66.9%) b	1.8° (20.1%) a
*Dinizia excelsa*	3.3° (49.8%) a	9.4° (79.6%) b	2.6° (51.5%) a
*Cedrelinga catenaeformis*	5.1° (71.7%) a	10° (84.9%) a	5.9° (46.0%) a
Overall Mean	7.1° (105%) ab	10.8° (72%) b	3.5° (72%) a

On the lines, different letters indicate statistically significant differences with 95% confidence level.

**Table 4:** Mean velocities (m·s^−1^) and coefficients of variation (%) obtained for polyhedral specimens with 26 faces of traditional dimensions (P26N), polyhedral specimens with 26 faces of reduced dimensions (P26A), and polyhedral specimens with 18 faces (P18). Species: *Apuleia leiocarpa*.

Velocities	*Apuleia leiocarpa*		
	P26N	P26A	P18
V_11_	5009 (3.6%) a	4864 (5.4%) a	5038 (3.9%) a
V_22_	2249 (2.6%) a	2258 (4.5%) a	2239 (7.2%) a
V_33_	1912 (3.6%) a	1916 (4.1%) a	1865 (6.4%) a
V_66_	1374 (2.0%) a	1385 (2.6%) a	1358 (3.0%) a
V_55_	1216 (3.3%) a	1211 (6.6%) a	1180 (6.3%) a
V_44_	913 (3.7%) a	933 (4.6%) a	907 (5.3%) a
V_12 45°_	1578 (5.2%) a	1637 (5.9%) a	1556 (3.1%) a
V_13 45°_	1406 (4.4%) b	1439 (1.5%) b	1344 (3.1%) a
V_23 45°_	964 (4.2%) a	947 (2.0%) a	963 (3.0%) a

On the lines, different letters indicate statistically significant differences with 95% confidence level.

**Table 5:** Mean velocities (m·s^−1^) and coefficients of variation (%) obtained for polyhedral specimens with 26 faces of traditional dimensions (P26N), polyhedral specimens with 26 faces of reduced dimensions (P26A), and polyhedral specimens with 18 faces (P18). Species: *Dinizia excelsa*.

Velocities	*Dinizia excelsa*		
	P26N	P26A	P18
V_11_	5137 (2.1%) a	5227 (4.9%) a	5183 (6.2%) a
V_22_	2232 (0.4%) a	2186 (2.6%) a	2188 (3.6%) a
V_33_	1868 (5.5%) a	1900 (6.3%) a	1896 (3.5%) a
V_66_	1382 (6.5%) a	1264 (2.7%) a	1387 (10.7%) a
V_55_	1166 (1.1%) a	1150 (3.3%) a	1140 (1.5%) a
V_44_	907 (0.3%) b	889 (2.7%) ab	886 (1.2%) a
V_12 45°_	1533 (2.5%) a	1484 (3.9%) a	1525 (1.9%) a
V_13 45°_	1349 (3.3%) a	1346 (3.2%) a	1348 (3.2%) a
V_23 45°_	937 (0.9%) ab	922 (1.1%) a	944 (2.1%) c

On the lines, different letters indicate statistically significant differences with 95% confidence level.

**Table 6:** Mean velocities (m·s^−1^) and coefficients of variation (%) obtained for polyhedral specimens with 26 faces of traditional dimensions (P26N), polyhedral specimens with 26 faces of reduced dimensions (P26A), and polyhedral specimens with 18 faces (P18). Species: *Cedrelinga catenaeformis*.

Velocities	*Cedrelinga catenaeformis*		
	P26N	P26A	P18
V_11_	5305 (8.7%) b	4478 (11.4%) a	4622 (13.1%) a
V_22_	2011 (1.9%) a	2098 (9.4%) a	2011 (1.9%) a
V_33_	1438 (10.5%) a	1529 (13.9%) a	1506 (11.3%) a
V_66_	1355 (3.0%) ab	1395 (1.9%) b	1331 (4.0%) a
V_55_	1092 (7.2%) a	1109 (3.5%) a	1127 (3.9%) a
V_44_	725 (4.3%) a	705 (3.1%) a	714 (5.2%) a
V_12 45°_	1512 (1.4%) a	1486 (7.2%) a	1484 (3.5%) a
V_13 45°_	1151 (5.7%) a	1185 (5.5%) a	1174 (5.3%) a
V_23 45°_	725 (4.3%) a	705 (3.1%) a	714 (5.2%) a

On the lines, different letters indicate statistically significant differences with 95% confidence level.

**Table 7:** Elastic parameters and coefficients of variation (%) obtained for polyhedral specimens with 26 faces of traditional dimensions (P26N), polyhedral specimens with 26 faces of reduced dimensions (P26A), and polyhedral specimens with 18 faces (P18). Species: *Apuleia leiocarpa*.

	P26N	P26A	P18
E_L_ (MPa)	16,136 (24.5%) a	16,252 (18.7%) a	15,749 (22.7%) a
E_R_ (MPa)	2574 (13.9%) a	2583 (9.6%) a	2599 (9.8%) a
E_T_ (MPa)	1904 (7.8%) a	1886 (8.5%) a	1808 (9.9%) a
G_RT_ (MPa)	673 (15.6%) a	704 (18.0%) a	662 (14.7%) a
G_LT_ (MPa)	1193 (15.8%) a	1191 (20.8%) a	1130 (20.4%) a
G_LR_ (MPa)	1514 (9.8%) a	1541(12.2%) a	1479 (9.7%) a
ν_RL_	0.10 (49.6%) a	0.08 (34.1%) a	0.11 (29.3%) a
ν_TL_	0.07 (39.2%) a	0.06 (35.0%) a	0.08 (36.1%) a
ν_LR_	0.62 (29.3%) a	0.51 (28.7%) a	0.62 (25.2%) a
ν_TR_	0.44 (4.3%) b	0.44 (1.9%) b	0.37 (11.7%) a
ν_LT_	0.58 (27.8%) a	0.49 (34.3%) a	0.71 (30.8%) a
ν_RT_	0.55 (14.9%) a	0.60 (4.98%) a	0.54 (13.2%) a

On the lines, different letters indicate statistically significant differences with 95% confidence level.

**Table 8:** Elastic parameters and coefficients of variation (%) obtained for polyhedral specimens with 26 faces of traditional dimensions (P26N), polyhedral specimens with 26 faces of reduced dimensions (P26A), and polyhedral specimens with 18 faces (P18). Species: *Dinizia excelsa*.

	P26N	P26A	P18
E_L_ (MPa)	21,767 (7.3%) a	22,303 (16.5%) a	22,107 (9.9%) a
E_R_ (MPa)	3346 (6.5%) a	3155 (8.4%) a	3293 (4.6%) a
E_T_ (MPa)	2446 (6.0%) a	2450 (5.5%) a	2519 (7.6%) a
G_RT_ (MPa)	918 (0.7%) b	849 (3.7%) a	864 (4.2%) a
G_LT_ (MPa)	1496 (4.1%) b	1422 (3.5%) a	1430 (3.0%) a
G_LR_ (MPa)	2111 (14.6%) a	1758 (6.0%) a	2132 (20.6%) a
ν_RL_	0.12 (15.3%) a	0.11 (19.7%) a	0.11 (20.3%) a
ν_TL_	0.07 (31.3%) a	0.08 (45.2%) a	0.08 (26.2%) a
ν_LR_	0.76 (18.4%) a	0.77 (21.2%) a	0.71 (16.1%) a
ν_TR_	0.41 (9.4%) a	0.44 (1.9%) a	0.41 (6.9%) a
ν_LT_	0.63 (25.3%) a	0.74 (18.6%) a	0.71 (32.2%) a
ν_RT_	0.55 (2.2%) a	0.55 (5.3%) a	0.53 (3.6%) a

On the lines, different letters indicate statistically significant differences with 95% confidence level.

**Table 9:** Elastic parameters and coefficients of variation (%) obtained for polyhedral specimens with 26 faces of traditional dimensions (P26N), polyhedral specimens with 26 faces of reduced dimensions (P26A), and polyhedral specimens with 18 faces (P18). Species: *Cedrelinga catenaeformis*.

	P26N	P26A	P18
E_L_ (MPa)	11,723 (10.0%) b	8323 (26.1%) a	10,182 (19.8%) ab
E_R_ (MPa)	1250 (21.2%) a	1163 (17.6%) a	1126 (17.0%) a
E_T_ (MPa)	624 (23.7%) a	604 (21.4%) a	620 (26.7%) a
G_RT_ (MPa)	183 (10.2%) a	239 (47.8%) a	229 (36.1%) a
G_LT_ (MPa)	602 (19.9%) a	618 (17.2%) a	643 (19.6%) a
G_LR_ (MPa)	921 (14.1%) a	967 (12.4%) a	884 (10.5%) a
ν_RL_	0.05 (22.7%) a	0.08 (37.4%) a	0.07 (27.2%) a
ν_TL_	0.05 (25.8%) a	0.05 (80.7%) a	0.05 (79%) a
ν_LR_	0.50 (22.1%) a	0.55 (29.0%) a	0.66 (38.5%) a
ν_TR_	0.39 (12.7%) a	0.44 (11.5%) a	0.44 (11.9%) a
ν_LT_	0.85 (14.2%) a	0.59 (47.3%) a	0.69 (36.8%) a
ν_RT_	0.79 (6.7%) a	0.84 (7.1%) a	0.84 (5.0%) a

On the lines, different letters indicate statistically significant differences with 95% confidence level.

## Data Availability

The data presented in this study are available on request from the corresponding author.
